# Hepatoprotective Effects of Nicotiflorin from *Nymphaea candida* against Concanavalin A-Induced and d-Galactosamine-Induced Liver Injury in Mice

**DOI:** 10.3390/ijms18030587

**Published:** 2017-03-08

**Authors:** Jun Zhao, Shilei Zhang, Shuping You, Tao Liu, Fang Xu, Tengfei Ji, Zhengyi Gu

**Affiliations:** 1 Key Laboratory for Uighur Medicine, Institute of Materia Medica of Xinjiang, Urumqi 830004, China; zhaojun21cn@163.com (J.Z.); xufangxj@163.com (F.X.); 2 School of Public Health, Xinjiang Medical University, Urumqi 830011, China; zhangsl6191@163.com (S.Z.); youshuping@163.com (S.Y.); 3 State Key Laboratory of Bioactive Substance and Function of Natural Medicines, Institute of Materia Medica, Chinese Academy of Medical Sciences and Peking Union Medical College, Beijing 100050, China

**Keywords:** nicotiflorin, *Nymphaea candida*, hepatoprotective, Concanavalin A, d-galactosamine

## Abstract

*Nymphaea candida* was used to treat hepatitis in Ugyhur medicine, and nicotiflorin (kaempferol 3-*O*-β-rutinoside) is the main characteristic component in this plant. In this study, The the hepatoprotective activities of nicotiflorin from *N. candida* were investigated by Concanavalin A (Con A, 20 mg/kg bw)- and d-Galactosamine (d-GalN, 800 mg/kg bw)-induced acute liver injury in mice. Pretreatment with nicotiflorin (25, 50, 100 mg/kg bw/day, p.o.) for ten days significantly reduced the impact of Con A toxicity (20 mg/kg bw) on the serum markers of liver injury, aspartate aminotransferase (AST), and alanine aminotransferase (ALT). The hepatic anti-oxidant parameters (malondialdehyde, MDA; superoxide dismutase, SOD; glutathione, GSH; and nitric oxide, NO) in mice with nicotiflorin treatment were significantly antagonized for the pro-oxidant effects of Con A. Moreover, pretreatment with nicotiflorin (100 mg/kg bw) significantly decreased Con A-induced elevation in the serum levels of pro-inflammatory cytokines interleukin-1β (IL-1β), interleukin-6 (IL-6), tumor necrosis factor-α (TNF-α), and interferon-γ (IFN-γ) (*p* < 0.05). A protective effect was reconfirmed against d-GalN-induced chemical liver injury, elevated serum enzymatic and cytokines levels were significantly decreased by nicotiflorin, and liver homogenate antioxidant indicators were significantly restored toward normal levels. Both histopathological studies also supported the protective effects of nicotiflorin. Therefore, the presented results suggest that nicotiflorin is the potent hepatoprotective agent that could protect the liver against acute immunological and chemical injury; this ability might be attributed to its antioxidant and immunoregulation potential.

## 1. Introduction

Liver, the major organ for the detoxification and metabolism of xenobiotics, is susceptibly injured by various factors such as toxic chemicals, excess consumption of alcohol, infections, and autoimmune disorders [1]. Moreover, liver injury is also a commonly pathological state of various liver diseases, and its long-term existence often leads to liver fibrosis, liver cirrhosis, and liver cancer [2]. Therefore, the prevention of liver injury is an important means of liver disease treatment [3]. It has always been one of focuses of pharmaceutical research to find significant hepatoprotective compounds from natural plants and traditional folk medicine. In recent years, more and more natural products with hepatoprotection have been isolated from various medicinal plants, such as silymarin, oleanolic acid, and curcumin [4,5,6].

**Figure 1:**
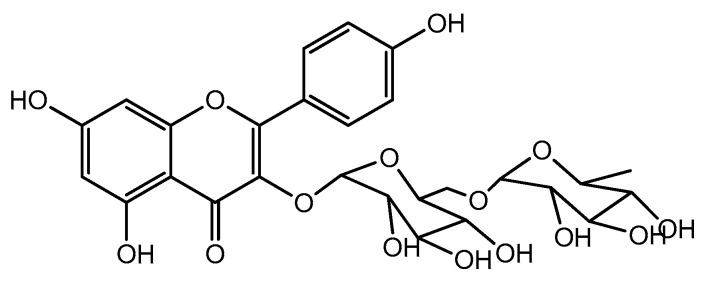
Chemical structure of nicotiflorin.

In Xinjiang, China, *Nymphaea candida* has been used as a folk medicine for head pains, coughs, hepatitis, and hypertension [7]. The previous study showed that extracts from the flowers of *N. candida* Presl have better free radical scavenging and hepatoprotective activities, and nicotiflorin was one of the main characteristic compounds in this plant [8]. Nicotiflorin (Figure 1), namely kaempferol 3-*O*-β-rutinoside, a flavonol glycoside isolated from a variety of plants (*Edgeworthia chrysantha*, *Carthamus tinctorius*, *N. candida*, etc.) [9,10,11], has been reported to have various pharmaceutical effects, such as antioxidant, anti-inflammatory, and neuroprotective effects [12,13,14,15]. In the previous study, nicotiflorin at the doses of 200 and 400 mg/kg bw showed preventive effects on CCl_4_-induced liver injury in mice [16]. This study aimed to investigate further the hepatoprotective effects of nicotiflorin (Doses as 25, 50 and 100 mg/kg bw) and its mechanisms by Concanavalin A (Con A)-induced and d-galactosamine (d-GalN)-induced liver injury in mice for the development and application of this compound as well as *N. candida*.

## 2. Results and Discussion

### 2.1. Protective Effect of Nicotiflorin on Con A Induced Hepatotoxicity in Mice

Concanavalin A (Con A), a lectin derived from jack bean seeds, has been widely used to establish an experimental murine model of hepatitis, and this model can mimic many pathological features of viral and autoimmune hepatitis in humans [17,18]. This reproducible liver injury is easily induced by a one-shot intravenous injection of Con A, and this damage could significantly increase the serum levels of transaminases as well as the filtration of neutrophils, macrophages, and T cells [19]. As a T cell mitogen, Con A can activate T cells to proliferate and produce pro-inflammatory cytokines including tumor necrosis factor-α (TNF-α), interferon-γ (IFN-γ), interleukin-1 (IL-1), and interleukin-6 (IL-6) [20].

**Figure 2:**
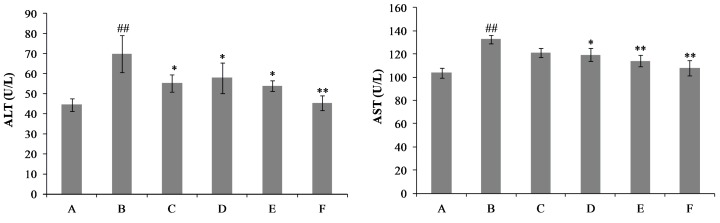
Effects of nicotiflorin on serum alanine aminotransferase (ALT) and aspartate aminotransferase (AST) in Con A- intoxicated mice. (A) Control group; (B) Con A-treated group; (C) Con A and biphenyl dicarboxylate (DDB, 150 mg/kg bw)-treated group; (D) Con A and nicotiflorin (25 mg/kg bw)-treated group; (E) Con A and nicotiflorin (50 mg/kg bw)-treated group; and (F) Con A and nicotiflorin (100 mg/kg bw)-treated group. Values are mean ± S.E.M., *n* = 10. ^##^ *p* < 0.01 compared with control group; * *p* < 0.05, ** *p* < 0.01 compared with Con A group.

The serum alanine aminotransferase (ALT) and aspartate aminotransferase (AST) activities are biochemical markers of liver damage [21]. Figure 2 shows that, after Con A injection, a statistically significant increasement in the serum ALT and AST levels was observed at 8 h, compared to the control group, as well as the liver index and spleen index (*p* < 0.01). After treatment with the drug for ten consecutive days, nicotiflorin at three different doses (25, 50 and 100 mg/kg bw) could remarkably prevent the Con A-induced increases of the serum activities of ALT and AST (*p* < 0.01, *p* < 0.05, Figure 2). Organ indexes of the liver and spleen were evaluated in mice. Compared with the Con A group, the elevated liver index and spleen index were also significantly reduced by nicotiflorin at different doses (25, 50, and 100 mg/kg bw) (*p* < 0.01, Table 1). Moreover, there were no significant differences in the changes of body weight before and after the experiment between each group (*p* > 0.05, Table 1).

**Figure 3:**
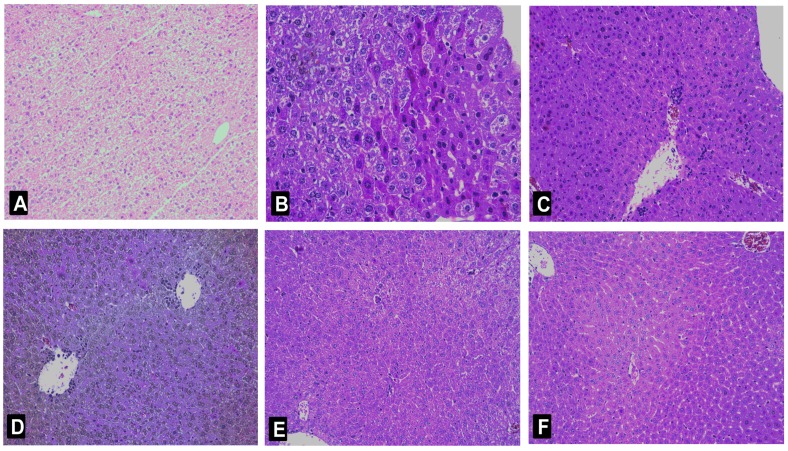
Histological analysis of the livers after Con A administration. Typical images were chosen from the different experimental groups (original magnification 10 × 20). (**A**) Control group; (**B**) Con A-treated group; (**C**) Con A and DDB (150 mg/kg bw)-treated group; (**D**) Con A and nicotiflorin (25 mg/kg bw)-treated group; (**E**) Con A and nicotiflorin (50 mg/kg bw)-treated group; and (**F**) Con A and nicotiflorin (100 mg/kg bw)-treated group.

Histological changes in liver tissues shown by hematoxylin-eosin (HE) staining confirmed the preventive effect of nicotiflorin against Con A-induced immunological liver injury (Figure 3). For livers in the control group, the extent of liver injury was grade 0; the hepatic lobule structure integrity, and a well-preserved cytoplasm, prominent nucleus, and nucleolus were shown (Figure 2A). After Con A injection, liver sections in the model group revealed extensive liver damage such as liver cells with severe edema, condensed nuclei, increased vacuole formation, acidophilic degeneration, inflammatory cells infiltration, centrilobular fatty changes, and widespread hepatocellular necrosis (grades III, Figure 3B). In contrast, mice pretreated with nicotiflorin (25, 50, and 100 mg/kg bw, Figure 3D–F), showed protective effects, and the injury scores of vacuole formation and hepatocellular necrosis were significantly decreased, with main liver damage grades of 0 and I (Table 2). The positive control drug, biphenyl dicarboxylate (DDB, 150 mg/kg bw), also significantly ameliorated liver damage induced by Con A (*p* < 0.05).

**Figure 4:**
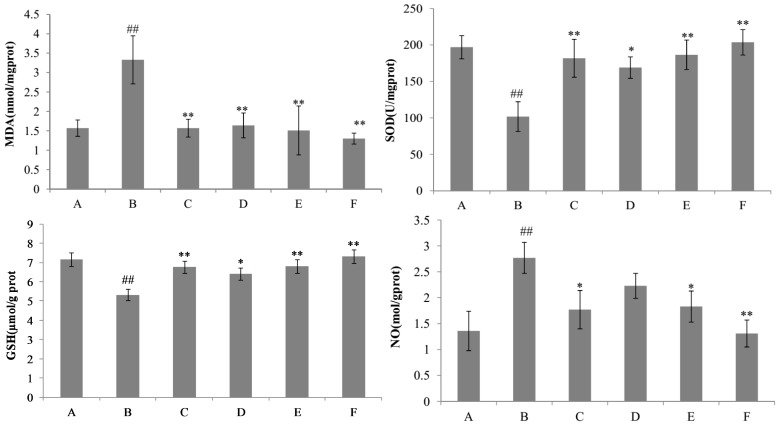
Effects of nicotiflorin on hepatic homogenate superoxide dismutase (SOD), malondialdehyde (MDA), glutathione (GSH), and nitric oxide (NO) in Con A-intoxicated mice. (A) Control group; (B) Con A-treated group; (C) Con A and DDB (150 mg/kg bw)-treated group; (D) Con A and nicotiflorin (25 mg/kg bw)-treated group; (E) Con A and nicotiflorin (50 mg/kg bw)-treated group; and (F) Con A and nicotiflorin (100 mg/kg bw)-treated group. Values are mean ± S.E.M., *n* = 10. ^##^ *p* < 0.01 compared with the control group; * *p* < 0.05, ** *p* < 0.01 compared with the Con A group.

Malondialdehyde (MDA), a major degradation product of lipid hydroperoxides, has attracted much attention as a indicator for assessing the extent of lipid peroxidation in oxidative liver damage. In this study, the hepatic level of malondialdehyde (MDA) was analyzed by the thiobarbituric acid (TBA) method [22]. Con A treatment markedly increased the hepatic MDA level compared with the control group, whereas the pre-administration of nicotiflorin (25, 50, and 100 mg/kg bw) significantly decreased the MDA levels (*p* < 0.05, Figure 4). The antioxidant (glutathione, GSH) content and antioxidant enzyme (superoxide dismutase, SOD) activity in the liver was also measured. The hepatic levels of GSH and SOD were conspicuously decreased in Con A-treated mice compared with those in the control group, whereas the pre-administration of nicotiflorin significantly reversed the decreased activities of GSH and SOD (*p* < 0.05, *p* < 0.01, Figure 4). Compared with control group, Con A injection significantly increased hepatic homogenate nitric oxide (NO) content (*p* < 0.01, Figure 4). Pretreatment with nicotiflorin (50 and 100 mg/kg) significantly decreased serum NO content (*p* < 0.05, *p* < 0.01, Figure 4). The positive control drug, DDB (150 mg/kg), also significantly decreased serum NO content (*p* < 0.05).

**Figure 5:**
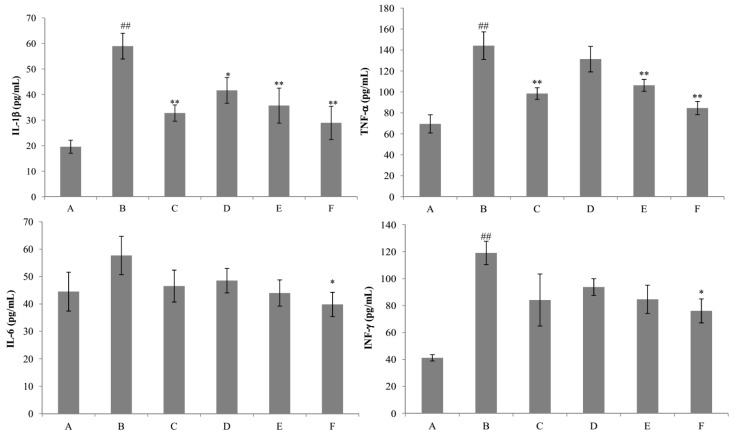
Effects of nicotiflorin on the serum interleukin-1β (IL-1β), tumor necrosis factor-α (TNF-α), interleukin-6 (IL-6), and interferon-γ (IFN-γ) in Con A-intoxicated mice. (A) Control group; (B) Con A-treated group; (C) Con A and DDB (150 mg/kg bw)-treated group. (D) Con A and nicotiflorin (25 mg/kg bw)-treated group; (E) Con A and nicotiflorin (50 mg/kg bw)-treated groupl and (F) Con A and nicotiflorin (100 mg/kg bw)-treated group. Values are mean ± S.E.M., *n* = 10. ^##^ *p* < 0.01 compared with the control group. * *p* < 0.05, ** *p* < 0.01 compared with the Con A group.

The hepatic natural killer T cells play important roles in Con A-induced liver injury by releasing a variety of cytokines, such as IFN-γ, TNF-α, IL-1β, and IL-6 [23]. Among the various cytokines released by Con A-activated T-cells, TNF-α and IFN-γ are considered to play critical roles in the development of massive hepatocellular apoptosis and necrosis [24]. In this study, compared with the Con A group, pretreatment with nicotiflorin at the middle and high doses (50 and 100 mg/kg bw) significantly decreased serum IL-1β (*p* < 0.01) and TNF-α (*p* < 0.01) levels, of which nicotiflorin at the low dose (25 mg/kg bw) also significantly decreased serum IL-1β levels (*p* < 0.05) (Figure 5). Moreover, nicotiflorin (100 mg/kg bw) could significantly decrease the elevated serum IFN-γ level by Con A (*p* < 0.01). The positive control drug, DDB, also significantly decreased serum IL-1β, TNF-α and IFN-γ levels compared to the Con A group (*p* < 0.05). Therefore, nicotiflorin might alleviate the uncontrolled immune response through immunomodulation to play a role of hepatoprotection.

### 2.2. Protective Effect of Nicotiflorin on d-GalN-Induced Hepatotoxicity in Mice

**Figure 6:**
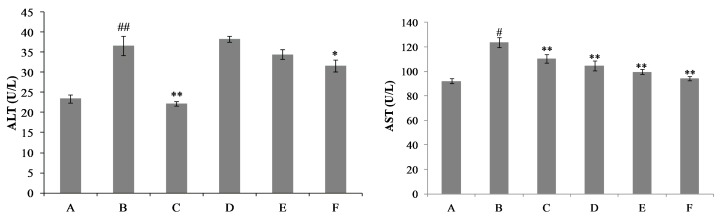
Effects of nicotiflorin on hepatic homogenate ALT and AST in d-GalN-intoxicated mice. (A) Control group; (B) d-GalN-treated group; (C) d-GalN and DDB (150 mg/kg bw)-treated group; (D) d-GalN and nicotiflorin (25 mg/kg bw)-treated group; (E) d-GalN and nicotiflorin (50 mg/kg bw)-treated group; and (F) d-GalN and nicotiflorin (100 mg/kg bw)-treated group. Values are mean ± S.E.M., *n* = 10. ^#^ *p* < 0.05, ^##^ *p* < 0.01 compared with the control group. * *p* < 0.01, ** *p* < 0.01 compared with the d-GalN group.

To further confirm the hepatoprotective activity of nicotiflorin, we investigated whether nicotiflorin protects against d-GalN-induced acute chemical liver injury. As a well-established hepatotoxicant, d-GalN can induce a liver injury similar to human viral hepatitis in its morphologic and functional features. Therefore, it is very useful for the evaluation of hepatoprotection to construct a liver injury model by d-GalN [25,26,27]. In this study, mice intoxicated with d-GalN developed severe hepatocellular injuries with a significant elevation in serum AST and ALT activities when compared to the control group (*p* < 0.01). Treatment with nicotiflorin at all doses (25, 50, 100 mg/kg bw) significantly prevented the elevation of serum AST compared to the d-GalN group (*p* < 0.01); and nicotiflorin at a high dose (100 mg/kg bw) significantly prevented the elevation of serum ALT compared to the d-GalN group (*p* < 0.05) (Figure 6). Moreover, nicotiflorin at all doses (25, 50, 100 mg/kg bw) significantly decreased the elevation of the liver index and speen index compared to the d-GalN group. The changes of body weight before and after the experiment did not show a significant difference between the groups (*p* > 0.05, Table 3).

**Figure 7:**
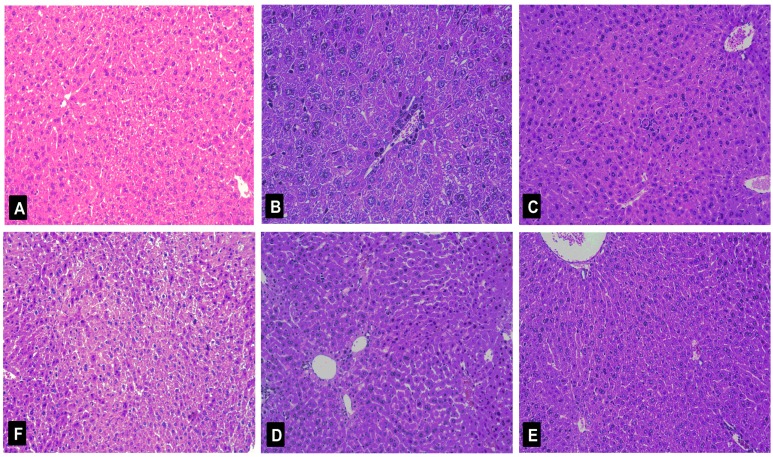
Histological analysis of the livers after d-GalN administration. Typical images were chosen from the different experimental groups (original magnification 10 × 20). (**A**) Control group; (**B**) d-GalN-treated group; (**C**) d-GalN and DDB (150 mg/kg bw)-treated group; (**D**) d-GalN and nicotiflorin (25 mg/kg bw)-treated group; (**E**) d-GalN and nicotiflorin (50 mg/kg bw)-treated group; and (**F**) d-GalN and nicotiflorin (100 mg/kg bw)-treated group.

In this study, we further examined liver histopathological characters to explore the protective effects of nicotiflorin on d-GalN-intoxicated mice. A photomicrograph of control mice liver showed the hepatic lobule structure integrity and the liver cells in mice to radiate out from central vein at the center (Figure 7A). A photomicrograph of d-GalN-intoxicated mice liver section showed swelling, loose cytoplasm, acidophilic degeneration, visible extensive hepatocytesteatosis, and lymphocytic infiltration (grade III, Figure 7B). As demonstrated in Table 4 and Figure 7D–F, nicotiflorin at different doses (25, 50, and 100 mg/kg bw) showed liver structure damage prevention effects at various levels against a d-GalN challenge. The histological observations basically supported the results obtained from biochemical index.

**Figure 8:**
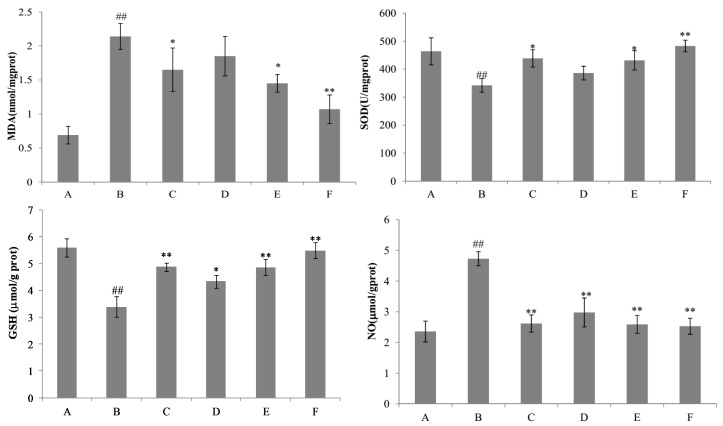
Effects of nicotiflorin on hepatic homogenate SOD, MDA, GSH, and NO in d-GalN-intoxicated mice. (A) Control group; (B) d-GalN-treated group; (C) d-GalN and DDB (150 mg/kg bw)-treated group; (D) d-GalN and nicotiflorin (25 mg/kg bw)-treated group; (E) d-GalN and nicotiflorin (50 mg/kg bw)-treated group; and (F) d-GalN and nicotiflorin (100 mg/kg bw)-treated group. Values are mean ± S.E.M., *n* = 10. ^##^ *p* < 0.01 compared with the control group. * *p* < 0.05, ** *p* < 0.01 compared with the d-GalN group.

**Figure 9:**
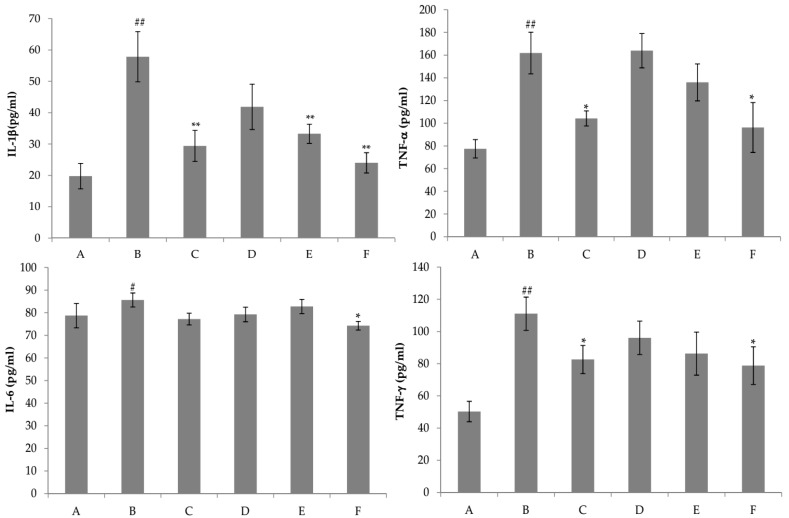
Effects of nicotiflorin on the serum IL-1β, TNF-α, IL-6, and IFN-γ in d-GalN-intoxicated mice. (A) Control group; (B) d-GalN-treated group; (C) d-GalN and DDB (150 mg/kg)-treated group; (D) d-GalN and nicotiflorin (25 mg/kg bw)-treated group; (E) d-GalN and nicotiflorin (50 mg/kg bw)-treated group; and (F) d-GalN and nicotiflorin (100 mg/kg bw)-treated group. Values are mean ± S.E.M., *n* = 10. ^#^ *p* < 0.05, ^##^ *p* < 0.01 compared with the control group. * *p* < 0.05, ** *p* < 0.01 compared with the d-GalN group.

Liver injury induced by d-GalN provoked a significant reduction of SOD and GSH activities (*p* < 0.01) and a significant increment of MDA and NO content (*p* < 0.01) in the liver homogenate of the d-GalN group as compared to the control group (Figure 8). The results showed that the content of GSH was significantly increased by nicotiflorin at the doses of 25, 50, and 100 mg/kg bw (*p* < 0.05, *p* < 0.01). Treatment with nicotiflorin (50 and 100 mg/kg bw) significantly prevented the reduction of SOD activity (*p* < 0.05, *p* < 0.01) and the increase of MDA content (*p* < 0.05, *p* < 0.01) induced by d-GalN intoxication. Compared with control group, Con A significantly increased hepatic homogenate NO content (*p* < 0.01). Nicotiflorin (25, 50, 100 mg/kg bw) could significantly decrease serum NO content compared to the d-GalN group (*p* < 0.01), and the positive control drug, DDB (150 mg/kg bw), also significantly decreased serum NO levels (*p* < 0.01). In the d-GalN group, the serum IL-1β, IL-6, TNF-α, and IFN-γ levels were significantly higher than that of the control group (*p* < 0.01). Treatment with nicotiflorin (50, 100 mg/kg bw) significantly reduced the increased serum IL-1β by d-GalN (*p* < 0.01). Moreover, compared with the d-GalN group, nicotiflorin (100 mg/kg bw) could significantly decrease serum TNF-α, IL-6 and IFN-γ levels (*p* < 0.05) (Figure 9).

## 3. Materials and Methods

### 3.1. Chemicals and Reagents

Concanavalin A (Con A) and d-galactosamine (d-GalN) were purchased from Sigma Chemical Co. (St. Louis, MO, USA). Biphenyl dicarboxylate (DDB) was obtained from Dezhou Deyao Pharmaceutical Co. Assay kits for aspartate aminotransferase (AST) and alanine aminotransferase (ALT) were provided by Zhongsheng Tech. (Beijing, China). Commercial kits used for determining (MDA), superoxide dismutase (SOD), glutathione (GSH), and nitric oxide (NO) activities were obtained from the Jiancheng Institute of Biotechnology (Nanjing, China). Elisa kits for interleukin-1β (IL-1β), interleukin-6 (IL-6), tumor necrosis factor-α (TNF-α), and interferon-γ (INF-γ) were supplied by Biosource Co. (St. Louis, MO, USA). All other chemicals were of analytical grade and were purchased from a local reagent retailer.

### 3.2. Plant Material and Preparation of Nicotiflorin

The dried flower buds of *N. candida* were purchased from the Traditional Uighur Medicine Hospital in Urumqi and identified by associate researcher Jiang He, Institute of Materia Medica of Xingjiang in China. Ten kilograms of this plant were extracted with 70% ethanol under reflux for 1 h three times, and 70% ethanol extracts was evaporated under vacuum. The 70% ethanol extracts were purified by D101 resin to obtain the extracts as follows: water and 30%, 50%, and 95% ethanol eluates, of which 50% of the ethanol eluates were applied to an ODS RP-18 column and eluted with mixtures of MeOH/H_2_O (0%–100%) successively. Nicotiflorin was obtained from 40% methanol eluates by Sephadex LH-20 chromatography repeatedly. The chromatographic analysis of nicotiflorin was performed using an high performance liquid chromatography (HPLC) system consisting of a Shimadzu LC-10ATvp and Phenomenex Gemini column (250 mm × 4.6 mm, 5 μm, with precolumn). The mobile phase was composed of A: 0.2% phosphoric acid aqueous solution and B: acetonitrile. The gradient program was set as follows; 0–5 min, 5% B in A; 5–10 min, 5%–11% B in A; 10–30 min, 11%–14% B in A; 30–60 min, and 14%–20% B in A. The detection wavelength was 266 nm, and the flow rate was 1.0 mL/min. The nicotiflorin purity content was quantified as 98.12% by peak area normalization method.

### 3.3. Animals

Kunming mice, weighing 20.0 ± 2.0 g, supplied by the Experimental Animal Centre of Xinjiang Medical University in China (No. SYXK(xin) 2011-0004). The mice were housed in plastic cages with a room temperature of 25 ± 1 °C under a 12 h light–dark cycle and were provided with rodent chow and water ad libitum. All procedures were approved by the Animal Care and Used Committee (No. 20150812-1; 12 August 2015) of Institute of Materia Medica of Xinjiang (Urumqi, China).

### 3.4. Concanavalin A (Con A)-Induced Hepatotoxicity

The mice were randomly divided into six groups; the control group, the Con A-induced liver injury model group, the positive control group (DDB, 150 mg/kg bw), and the nicotiflorin groups (25, 50, and 100 mg/kg bw). Mice in the control and model groups were given distilled water by intragastric administration (ig) (0.2 mL/10 g bw, once daily). Mice in the DDB and nicotiflorin groups received DDB (150 mg/kg bw, ig, once daily) and nicotiflorin (25, 50, and 100 mg/kg bw, ig, once daily), respectively. All administrations were conducted for ten consecutive days. One hour after the last administration on the seventh day, mice in the control group received saline (0.1 mL/10 g bw, iv) while mice in the other groups were injected with Con A (20 mg/kg bw) [28]. Mice were sacrificed after fasting for 8 h, blood samples were collected, and serum was isolated for further tests; the livers were removed for biochemical studies and histopathological analysis.

### 3.5. d-Galactosamine (d-GalN)-Induced Hepatotoxicity

To study the effect of nicotiflorin on d-GalN-induced liver injury, mice were randomly divided into six groups with 10 mice per group as follows; control group, model group, DDB group (150 mg/kg bw), and the nicotiflorin groups (25, 50 or 100 mg/kg bw). The mice in the pre-treatment groups were administered by intragastric gavage (0.1 mL/10 g bw) with different doses of nicotiflorin, respectively, once a day for 7 days, while the control and model groups were given distilled water only. On the seventh day, 1 h after of the last administration, mice in various groups were given an intraperitoneal injection of d-GalN in normal saline (800 mg/kg, 0.2 mL/10 g b.w.), while the control group was injected intraperitoneally with an equal amount of normal saline solution. Mice were sacrificed after fasting for 8 h. Blood samples were collected and serum was isolated for further tests. The livers were removed for biochemical studies and histopathological analysis [29].

### 3.6. Measurement of Liver Index, Spleen Index and Body Weight in Mice

The body weight of the animals was weighed before and after the experiment. The liver index and spleen index were calculated as liver weight (mg) and spleen weight (mg) divided by the body weight of the mice (10 g), respectively.

### 3.7. Measurement of Aminotransferase and Cytokine Levels in the Serum

The blood samples were collected by retroorbital bleeding, the collected blood was centrifuged at 3000 r/min for 10 min and 4 °C, and serum was obtained. The activities of serum enzymes alanine aminotransferase (ALT) and aspartate aminotransferase (AST) were determined using the commercial assay kits. Enzyme activities were expressed as an international unit (U/L). The levels of IL-1β, IL-4, TNF-α, and IFN-γ in plasma were determined using enzyme-linked immunosorbent assay (ELISA) kits according to the kit introduction.

### 3.8. Measurement of Liver Homogenate Contents of MDA, SOD, GSH and NO

Liver samples were homogenized in normal saline to give a 10% (*w*/*v*) liver homogenate and then centrifuged at 3000 rpm for 10 min at 4 °C. Supernatant was used to determine the MDA, GSH, SOD, and total protein concentrations by using the detection kits according to the manufacturer’s protocols. The levels of NO in liver homogenate were measured using nitrate reductase assay according to the kit introduction.

### 3.9. Histopathological Examination

For the histological investigations, liver tissues were removed from a portion of the left lobe, fixed in 10% formalin, embedded in paraffin, sliced in 5 μm sections, and stained with hematoxylin and eosin (H and E) according to standard protocols. The slides were observed for conventional morphological evaluation under a light microscope (Olympus BX43, Olympus, Tokyo, Japan) and photographed at 10 × 20 magnification. The degree of liver histological damage was scored as follows on a scale of 0–III: grade 0, no necrosis with normal liver tissue structure; I, part of the liver tissue swelling accompanied with sporadic dotted necrosis and necrosis <1/4 of the hepatic lobule area; grade II, liver cell swelling, visible in the spotty necrosis and minimal necrosis, inflammatory cells infiltration in the portal area, and necrosis <1/2 of the hepatic lobule area; III, liver cell swelling, massive necrosis, inflammatory cell infiltration, and necrosis >1/2 of the hepatic lobule area [30].

### 3.10. Statistical Analysis

All data were expressed as the mean ± standard error (S.E.M.). The differences between different groups were analyzed using one-way analysis of variance (ANOVA) (SPSS software package for windows, version 13.0, Chicago, IL, USA). * *p* < 0.05 and ** *p* < 0.01 were taken as statistically significant.

## 4. Conclusions

The present study clearly demonstrates that nicotiflorin possess significant *in vivo* anti-hepatotoxic activities, and this is confirmed in two experimental animal models. Therefore, nicotiflorin could be used as a protective agent against acute liver injury, and its mechanism might be attributed to its antioxidant and immunoregulatory capacities.

## Figures and Tables

**Table 1:** Effects of nicotiflorin on the body weight and liver, speen index in Concanavalin A (Con A)-intoxicated mice.

Group	Inital BW (g)	Final BW (g)	Liver Index	Speen Index
Control	19.83 ± 0.37	26.49 ± 0.39	48.12 ± 0.80	3.65 ± 0.18
Con A	20.41 ± 0.49	27.44 ± 0.65	79.59 ± 2.20 ^##^	7.19 ± 1.42 ^##^
DDB (150 mg/kg bw) + Con A	20.01 ± 0.53	26.53 ± 0.57	55.82 ± 0.63 **	5.01 ± 0.22 **
nicotiflorin (25 mg/kg bw) + Con A	19.84 ± 0.47	26.64 ± 0.40	61.82 ± 1.22 **	5.48 ± 0.27 **
nicotiflorin (50 mg/kg bw) + Con A	20.11 ± 0.70	27.09 ± 0.41	57.39 ± 2.15 **	4.89 ± 0.36 **
nicotiflorin (100 mg/kg bw) + Con A	21.69 ± 0.31	28.18 ± 0.37	53.98 ± 1.13 **	4.24 ± 0.20 **

Values are mean ± S.E.M., *n* = 10; ^##^ *p* < 0.01 compared with control group; ** *p* < 0.01 compared with Con A group; BW, body weight.

**Table 2:** Effects of nicotiflorin on the pathological grading of Con A-intoxicated mice.

Group	0	I	II	III	*p*-Value
Control	10	0	0	0	-
Con A	0	0	2	8	^#^
DDB (150 mg/kg bw) + Con A	1	5	2	2	*
nicotiflorin (25 mg/kg bw) + Con A	2	6	1	1	*
nicotiflorin (50 mg/kg bw) + Con A	2	5	2	1	*
nicotiflorin (100 mg/kg bw) + Con A	2	6	1	1	*

*n* = 10; ^#^ *p* < 0.01 compared with control group; * *p* < 0.05, compared with Con A group.

**Table 3:** Effects of nicotiflorin on the body weight and the liver and speen indexes in d-GalN-intoxicated mice.

Group	Inital BW (g)	Final BW (g)	Liver Index	Speen Index
Control	20.72 ± 0.59	25.02 ± 1.11	45.12 ± 1.13	4.25 ± 0.22
d-GalN	20.28 ± 0.34	26.35 ± 0.72	84.44 ± 1.47 ^##^	8.26 ± 0.20 ^##^
DDB (150 mg/kg bw) + d-GalN	21.36 ± 0.42	26.38 ± 0.58	53.75 ± 1.09 **	5.54 ± 0.18 **
nicotiflorin (25 mg/kg bw) + d-GalN	21.25 ± 0.48	25.64 ± 0.46	65.44 ± 0.89 **	6.43 ± 0.57 **
nicotiflorin (50 mg/kg bw) + d-GalN	21.26 ± 0.59	26.13 ± 0.80	57.48 ± 2.46 **	5.55 ± 0.30 **
nicotiflorin (100 mg/kg bw) + d-GalN	20.71 ± 0.59	26.75 ± 0.56	49.35 ± 1.50 **	4.88 ± 0.23 **

Values are mean ± S.E.M., *n* = 10. ^##^ *p* < 0.01 compared with the control group. ** *p* < 0.01 compared with the Con A group. BW, body weight.

**Table 4:** Effects of nicotiflorin on the pathological grading of d-GalN-intoxicated mice.

Group	0	I	II	III	*p*-Value
Control	10	0	0	0	-
d-GalN	0	0	3	7	^#^
DDB (150 mg/kg bw) + d-GalN	1	6	2	1	*
nicotiflorin (25 mg/kg bw) + d-GalN	1	5	3	1	*
nicotiflorin (50 mg/kg bw) + d-GalN	2	4	3	1	*
nicotiflorin (100 mg/kg bw) + d-GalN	3	5	1	1	*

*n* = 10; ^#^ *p* < 0.01 compared with control group; * *p* < 0.05, compared with d-GalN group.
